# The deleterious effects induced by an acute exposure of human skin to common air pollutants are prevented by extracts of *Deschampsia antarctica*

**DOI:** 10.1038/s41598-021-03190-2

**Published:** 2021-12-09

**Authors:** Sandra Fernández-Martos, María I. Calvo-Sánchez, Ana Lobo-Aldezabal, Ana Isabel Sánchez-Adrada, Carmen Moreno, María Vitale, Jesús Espada

**Affiliations:** 1 Experimental Dermatology and Skin Biology Group, Ramon y Cajal Institute for Health Research (IRYCIS), Ramón y Cajal University Hospital, Madrid, Spain; 2 Biosciences Research Institute, School of Experimental Sciences, Universidad Francisco de Vitoria, UFV, Building E, Ctra. M-515 Pozuelo-Majadahonda Km 1,800, 28223 Pozuelo de Alarcón, Madrid, Spain; 3 Medical Affairs Department, Cantabria Labs, Madrid, Spain; 4 Anatomic Pathology Service, San José and Santa Adela Red-Cross Central Hospital, Madrid, Spain; 5 Anatomic Pathology Service, Ramón y Cajal University Hospital, Madrid, Spain; 6 Centro Integrativo de Biología y Química Aplicada (CIBQA), Universidad Bernardo O’Higgins, Santiago, Chile

**Keywords:** Cell biology, Drug discovery

## Abstract

The homeostatic and regenerative potential of the skin is critically impaired by an increasing accumulation of air pollutants in human ecosystems. These toxic compounds are frequently implicated in pathological processes such as premature cutaneous ageing, altered pigmentation and cancer. In this scenario, innovative strategies are required to tackle the effects of severe air pollution on skin function. Here we have used a Human Skin Organotypic Culture (HSOC) model to characterize the deleterious effects of an acute topic exposure of human skin to moderately high concentrations of common ambient pollutants, including As, Cd, Cr, dioxins and tobacco smoke. All these toxic compunds inflict severe damage in the tissue, activating the AHR-mediated response to xenobiotics. We have further evaluated the potential of an aqueous leaf extract of the polyextremophile plant *Deschampsia antarctica* (Edafence) to protect human skin against the acute exposure to toxic pollutants. Our results indicate that pre-treatment of HSOC samples with this aqueous extract conuterbalances the deleterious effects of the exposure to toxic comunds and triggers the activation of key genes invoved in the redox system and in the pro-inflammatory/wound healing response in the skin, suggesting that this natural compound might be effectively used in vivo to protect human skin routinely in different daily conditions.

## Introduction

The skin is the largest organ of the human body and constitutes an essential defensive barrier against external pathogens and physical–chemical damage involving thermal deregulation, dehydration, chemical attack and solar (including ultraviolet) radiation^1,2^. To deal with these critical challenges, the skin exhibits a high self-renewal and regenerative potential, driven by very dynamic stem cell niches^1^. This feature is combined with a highly specialized immune response system that is quickly activated upon structural damage, involving wound and burn healing processes, or pathogen invasion^2–4^. Equally important, the skin is extensively innervated by a complex neuroendocrine network that supports the integration of a very precise sensing of different external and internal environmental signals with specific homeostatic responses at local and systemic levels^5^.

As with all tissues and organs in higher eukaryotes, the viability of mammalian skin function is gradually compromised by the natural ageing process. In addition, in current human ecosystems, characterized in most cases by a sustained increase of life expectancy and a rapid environmental accumulation of human-derived bio-toxic waste materials, the cutaneous homeostatic and regenerative potential is also being critically impaired in an accelerated way by a concomitantly increasing concentration of several air pollutants. These toxic compounds are directly implicated in skin pathological processes such as premature ageing, atopic dermatitis, psoriasis, altered pigmentation, chronic wounds and cancer. Metal and metalloid compounds are among the most common air pollutants as main components of ambient particulate matter. As air pollutants these compounds not only impair heavily the respiratory tract but may also affect skin function and integrity. In particular, toxic Arsenic, Cadmium and Chromium compounds, for the most part released to the atmosphere as a result of anthropogenic activity, may have a significant impact on human skin.

Toxic Arsenic compounds, in the form of interconvertible trivalent, As(III), or pentavalent, As(V), sulfide salts, are ubiquitous. Most of the human-derived air Arsenic emissions are the result of metal smelters, fuel combustion and the use of pesticides^6^. Arsenic compounds promote the intracellular generation of ROS and subsequent oxidative stress in parallel with the allosteric inhibition of pyruvate dehydrogenase, impairment of glycolysis, oxidative phosphorylation and mitochondrial dysfunction. All these severe biochemical malfunctions ultimately result in the shutdown of ATP production, induction of cell death and multi-system organ failure^7,8^. At a systemic level, acute massive exposure to Arsenic compounds promotes muscle cramps and convulsions, headaches and mental confusion, stomach pain, diarrhea and vomiting, that may finally cause death^6,7^. Chronic exposure to low Arsenic levels is related to different types of cancer, cardiovascular, cerebrovascular and respiratory diseases and diabetes^6–9^. With respect to the skin, it has been extensively reported an association of a chronic cutaneous exposure to Arsenic compounds with hyperkeratosis and different types of cancer, including basal cell carcinoma, squamous cell carcinoma and carcinoma in situ^10^; also an acute exposure of the skin to these compounds has been associated with the rapid emergence of pre-cancerous lesions and other cutaneous pathological conditions, including hair loss, hyperpigmentation and thickening of the tissue^6,10,11^.

Toxic Cadmium compounds, in the form of metallic or divalent, Cd(II), oxide salts, are mainly released into the atmosphere as a consequence of anthropogenic activity derived from metal production (copper, zinc, cadmium, iron, steel), refuse incineration (plastics, nickel–cadmium batteries), and combustion of coal and oil^12,13^. Cadmium compounds may induce a severe oxidative stress in cells and tissues, by promoting ROS production, glutathione depletion and the inhibition of key antioxidant enzymes, including catalases and dismutases, and the oxidative phosphorylation chain in the mitochondria^14–17^. Cadmium compounds also impair cell proliferation and differentiation programs, inducing DNA mutations and subsequent genome instability, ultimately promoting carcinogenesis or pathological cell death ^14,16,17^. The toxic effects of Cadmium compounds in different organs and tissues, including the skeletal (e. g. Itai-itai disease), respiratory, cardiovascular and reproductive systems kidney and liver has been also extensively described^18,19^. It has been also reported that a continuous exposure of the skin to Cadmium pollutants may cause hyperkeratosis, acanthosis and ulcerative lesions^12,18^.

Toxic Chromium compounds, in the form of hexavalent, Cr(VI), chromates, mainly oxide and potassium salts, are released into the environment for the most part as a consequence of human activity during the manufacturing of dyes, paints, cements, mortar, leather tanning and anti-corrosive products for automobile and aerospace industry. The effects of Cr(VI) as a potent carcinogenic, genotoxic and hemotoxic agent has been extensively reported^8,9,20,21^*.* Once incorporated into cells and tissues, Cr(VI) is rapidly reduced to Cr(III), an otherwise essential trace element for mammals. This redox reaction yields highly reactive chemical intermediates, including Cr(V), Cr(IV) and reactive oxygen radicals that can irreversible react with lipid, protein and nucleic acids further inducing pathological cell death or carcinogenic processes^8,9^. At a systemic level, acute Chromium poisoning usually occurs by ingestion while chronic exposure occurs by continuous inhalation or cutaneous exposure. Acute ingestion causes fever, gastrointestinal ulceration/corrosion, nausea, diarrhea, vomiting, muscle cramps, severe renal and liver damage, intravascular haemolysis and vascular system collapse, and may result in multisystem organ failure and death^22,23^. Chronic inhalation causes bronchitis, rhinitis, sinusitis and lung cancer. In the skin and other epithelial tissues a chronic Chromium exposure may promote penetrating ulcers, incapacitating eczematous and edematous dermatitis, keratitis in the eye cornea, gingivitis, periodontitis, nasal ulcers and irritation of the conjunctiva and mucous membranes^20,24^.

Dioxins and tobacco smoke constitute also an important fraction of the anthropogenic-derived air pollutant loaded into the atmosphere showing important deleterious effects on skin physiology. The primary and most toxic dioxin molecule is 2,3,7,8-tetrachlorodibenzo para-dioxin (TCDD). The generic name “dioxins” is further used to describe a family of structurally related, usually toxic molecules, encompassing polychlorinated dibenzo para-dioxins (PCDDs), polychlorinated dibenzofurans (PCDFs) and dioxin-like polychlorinated biphenyls (PCBs)^25^.These compounds are mostly released into the atmosphere as by-products of industrial processes including chlorine bleaching of paper pulp, smelting, manufacturing of herbicides and pesticides, and waste incineration^26,27^. Dioxins are ubiquitous and persistent environmental pollutants (POPs) of human ecosystems and are accumulated in the food chain, mainly in fatty acids, meat, fish, selfish and dairy products^28^. The toxicity of dioxins relies on the number and position of chlorine atoms and all of them share the same mechanisms of action involving the activation of the Aryl hydrocarbon Receptor (AhR receptor)^29–32^. Dioxins are harmful carcinogens, and long-term exposure to these compounds is associated with carcinogenesis and with the severe impairment of immune, endocrine, nervous and reproductive systems^33^. Acute, short-term exposure to high dioxin levels may cause immediate hepatitis, pancreatitis and severe skin lesions such as chloracne and hyperpigmentation^33–35^.

Tobacco smoke is an aerosolised by-product of tobacco combustion during the smoking of cigarettes and related products. This aerosol contains several potentially hazardous and tumorigenic compounds, including carbon monoxide, hydrogen cyanide, polycyclic aromatic hydrocarbons (e. g. benzopyrene), aldehydes (e. g. formaldehyde), aromatic amines (e. g. nicotine), N-nitrosamines, nitrogen oxide, benzene, toluene, together with arsenic, cadmium, chromium and dioxins^36^. Chronic exposure to tobacco smoke has been associated with different pathological conditions including lung cancer, emphysema, chronic obstructive pulmonary disease, bronchitis, pneumonia, asthma and cardiovascular disorders^37–40^. The relationship between tobacco smoke, premature skin ageing and hair loss has been previously reported^41,42^. Also, it has been reported that tobacco smoke components interfere with the synthesis of collagens and activate dermal matrix metalloproteases further promoting the degradation of collagens, elastin and proteoglycans in the dermis^41^, a hallmark of skin ageing.

In this scenario, different innovative strategies to tackle efficiently the effects of everyday exposure to heavy air pollution on skin function are currently under development. A promising approach comprises the screening and evaluation of extracts/compounds derived from extremophile organisms, particularly plants. These organisms exhibit vigorous growth and viability under extreme environmental conditions of temperature, salinity, pressure, water availability, solar irradiation, etc.^43^. Extremophile plants can thrive in ecosystems showing harsh environmental characteristics, including constitutive drought, extreme cold or pH and other stressors^44–46^. The exceptional ability of these plants to survive in extreme environments is dictated by the particular evolution of the corresponding genomes^44^.

Here we have evaluated the effects of an acute exposure of human skin to common air pollutants and the potential of an aqueous leaf extract of *Deschampsia antarctica* (Edafence)^47–49^*,* to prevent the deleterious effects of these pollutants on the skin. This plant is a polyextremophile Gramineae native to Antarctica capable of grow under extreme conditions of solar irradiation, temperature, dryness, salinity and oxidative stress. Such prominent resilience to extreme environmental conditions rest upon unique, evolutionary shaped, molecular mechanisms providing highly efficient protection against environmental aggression, particularly the ability to synthesize high ammounts of antioxidant compounds derived from the phenylpropanoid pathway, including phenolic acids and flavonoids^50–53^. In this sense, we have previously reported that Edafence exhibits a significant protective effect in skin fibroblast cultures exposed to deleterious concentrations of hydrogen peroxide or blue light^47–49^*,* suggesting the potential of this extract to prevent cellular damage induced by ambient pollutants.

## Results and discussion

In a step forward, here we have used a Human Skin Organotypic Culture (HSOC) model to characterize in detail the morphofunctional effects of an acute exposure of human skin to common air pollutants, including toxic metals (As, Cr, Cd), dioxins and tobacco smoke. Typically, these organotypic skin samples can be maintained in culture up to 7–9 days showing a morphological profile closely resembling normal human skin in native conditions. Taking into account this timeframe, we have decided to analyse the effects of an acute exposure (24 h) of freshly established HSOCs to moderately high concentrations of toxic pollutants and further characterization 48 h later. We consider that these experimental conditions may provide very useful information on the effects of ambient pollutants in human skin. We have evaluated different morphological parameters as well as changes in the localization/expression patterns of cell death and proliferation markers, and components of signaling pathways implicated in toxic response and inflammation. We have also evaluated the potential of (Edafence)*,* to prevent the deleterious effects of an acute exposure of HSCOs to air toxic pollutants.

**Figure 1:**
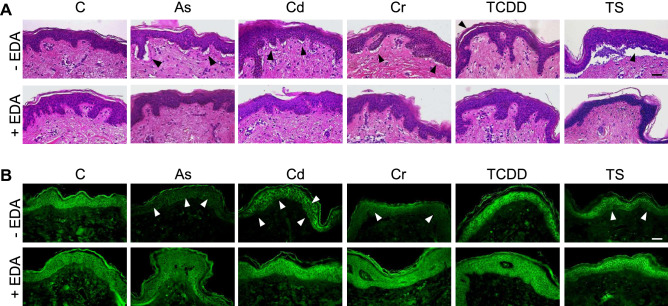
*Deschampia antarctica* aqueous extract (Edafence) efficiently prevents the structural tissue damage induced by acute exposure of Human Skin Organ Cultures to common ambient pollutants. HSOC histological sections from control samples (C) or samples exposed to As(III), Cd(II), Cr(VI), 2,3,7,8-tetrachlorodibenzo-p-dioxin (TCDD) or Tobacco Smoke culture medium infusions (TS) pre-treated or not with *Deschampia antarctica* aqueous extracts (EDA) showing (**A**) overall tissue morphology after H&E staining (black arrowheads point to major structural alterations in the tissue) and (**B**) fluorescent immunolocalization of the E-cadherin epidermal cell–cell adhesion marker (white arrowheads highlight representative areas of protein downregulation/delocalization in the tissue). Images are representative of at least three different samples for each experimental condition. Bars: 100 µm.

Acute exposure of HSOC samples to different metal pollutants, As(III), Cd(II), Cr(VI)), induces similar severe structural alterations in the tissue mainly revealed as an extensive epidermal-dermal detachment in a local ridge-patch pattern (Fig. 1A). Exposure of HSOC samples to high concentrations of tobacco smoke culture medium infusions (TS) results in a complete epidermal-dermal detachment. In addition, an inflammatory infiltrate appears between dermis and epidermis, and a slight edema is also registered in the epidermis (Fig. 1A). Also, acute exposure of HSOC samples to 2,3,7,8-tetrachlorodibenzo-p-dioxin (TCDD) promotes a significant desquamation of the stratum corneum (Fig. 1A). All these noticeable morphological alterations, sufficient in all cases to compromise decisively tissue functionality, are efficiently prevented by pre-treatments of HSCOs with *Deschampia antarctica* aqueous leaf extracts (Fig. 1A). These observations indicate the existence of a compound, or group of compunds, in this extremophile plant that are able to efficiently protect HSOC sample against acute pollutant exposure and/or promote rapid tissue healing. It is to note that no significant changes in epidermal thickness, number of epidermal papillae, collagen/elastin network density or morphology, fibroplasia or hyperpigmentation were observed in HSOC samples exposed to different toxic pollutants as compared to control samples, pre-treated or not with Edafence.

Intriguingly, HSOC exposure to each particular ambient pollutant affects in a different way the expression/localization of the E-cadherin homotypic cell–cell adhesion receptor, a crucial marker of epidermal integrity. Thus, acute exposure to As(III) and Cr(VI) promotes an extensive downregulation of E-cadherin expression in all epidermal layers, exposure to Cd(II) induces a partial downregulation and delocalization of E-cadherin in cells of all epidermal layers, specially ubiquitous in the basal layer, while exposure to TS infusions results in an extensive loss of the adhesion receptor restricted to basal and spinous layers in an otherwise detached epidermis (Fig. 1A). Notably, all these alterations were almost completely prevented by pre-treatments of the tissue with *Deschampia antarctica* aqueous leaf extracts (Fig. 1B). By contrast, exposure of HSOCs to TCDD does not alter significantly the expression or localization of E-cadherin in the tissue (Fig. 1B). These differences probably reflect the diversity of the molecular mechanisms involved in or affected by the exposure of HSOCs to each particular ambient pollutant.

**Figure 2:**
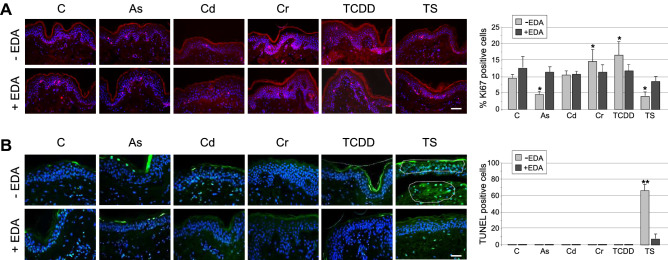
*Deschampia antarctica* aqueous extract (Edafence) counteract the alterations in cell proliferation patterns induced by acute exposure of Human Skin Organ Cultures to common ambient pollutants. HSOC histological sections from control samples (C) or samples exposed to As(III), Cd(II), Cr(VI), 2,3,7,8-tetrachlorodibenzo-p-dioxin (TCDD) or Tobacco Smoke culture medium infusions (TS) pre-treated or not with *Deschampia antarctica* aqueous extracts (EDA) showing (**A**) the fluorescent localization of the proliferation marker Ki-67 (left panel; bar: 100 µm) together with the quantification of the % of Ki-67 positive cells in each sample group (right panel), and (**B**) the fluorescent localization of apoptotic cells by the TUNEL assay (left panel, dotted lines delimit areas of a high TUNEL-positive cell content; bar: 50 µm) together with the quantification of the % of TUNEL positive cells in each sample group (right panel). Images are representative of at least three different samples for each experimental condition. For quantifications, the ratio of positive cells for the indicated proteins in histological sections of HSOC samples was calculated as described in the Methods section. The mean + SD of the positive cell average (%) in three different samples for each experimental condition is represented (*, significant *p* ≤ 0.1; **, significant *p* ≤ 0.05).

In this sense, acute exposure of HSOC samples to different ambient pollutants results also in different alterations of the cell proliferation pattern in the tissue, evaluated as the cellular index for the Ki67 marker, a non-histone nuclear protein highly expressed during the active phase of the cell cycle from late G1 to S and G2. Thus, acute exposure to As(III) or TS promotes a marked reduction in the number of Ki67 positive cells, while exposure to Cr(VI) and TCDD induces a significant increase in the number of proliferating cells (Fig. 2A). However, it is noteworthy that, in all cases, pre-treatment of HSOC samples with *Deschampia antarctica* aqueous extracts restore cell proliferation patterns to normal levels (Fig. 2A). These observations indicate that active compounds in Edafence may counteract both positive and negative imbalances of cell growth in the tissue, suggesting a direct regulatory effect on the integrated molecular circuit controling cell proliferation in human skin.

We have also evaluated the potential activation of cell death programs induced after an acute exposure of HSOC samples to ambient pollutants, quantified as the index of TUNEL assay positive cells in the tissue. We have found that, as a rule, none of the toxic compounds, in the experimental conditions tested in this study, promote a noticeable increase in the number of dead cells (Fig. 2B). Only in the case of TS acute exposure a strong increase in the cell death index is oberved in HSOC samples, and this increase is significantly prevented by pre-treatment with *Deschampia antarctica* aqueous leaf extracts (Fig. 2B). This fact suggest that the type and degree of tissue damage induced by an acute exposure of HSOCs to the toxic compounds used here, and in the concentration and time ranges described, is not in the scope of the molecular mechanisms able to trigger programmed cell death signaling pattway.

**Figure 3:**
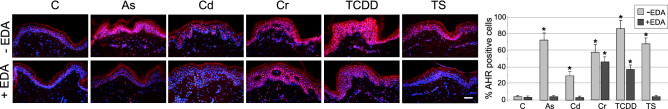
*Deschampia antarctica* aqueous extract (Edafence) revert the overexpression of the Aryl Hydrocarbon Receptor (AhR) induced by acute exposure of Human Skin Organ Cultures to common ambient pollutants. HSOC histological sections from control samples (C) or samples exposed to As(III), Cd(II), Cr(VI), 2,3,7,8-tetrachlorodibenzo-p-dioxin (TCDD) or Tobacco Smoke culture medium infusions (TS) pre-treated or not with *Deschampia antarctica* aqueous extracts (EDA) showing the fluorescent immunolocalization of the AhR protein. Images are representative of at least two different samples for each experimental condition. For quantifications, the ratio of positive cells for the indicated proteins in histological sections of HSOC samples was calculated as described in the Methods section. The mean + SD of the positive cell average (%) in two different samples for each experimental condition is represented (*, significant *p* ≤ 0.05). Bar: 100 µm.

Next we have investigated changes in the intracellular expression/localization of the AhR in response to acute exposure of HSOCs to ambient pollutats. The AhR is a transcription factor activated by ligand binding that regulates the coordinated expression of different enzymes implicated in xenobiotics metabolism and is considered as a key molecular indicator of the response of mammalian cells and tissues to ambient pollutants exposure^30–32^. Our resuts show that acute exposure of HSOCs to As(III), Cr(VI), TCDD or TS induces a strong expression of AhR mostly restricted to all epidermal layers (Fig. 3). This response, although noticeble, is moderate in the case of Cd(II) exposure (Fig. 3). These results confirm that the global gene expression response to toxic metals/xenobiotics that is coordinated by AhR is also presumably triggered in the HSOC model after acute exposure to the ambient pollutants used in this study. Pre-treatment of HSOC samples with *Deschampia antarctica* aqueous leaf extracts before pollutant exposure significantly prevents AhR overexpression in most cases except for Cr(VI) (Fig. 3), suggesting that acute exposure of the tissue to this particular toxic metal requires a specific sustained response of the tissue that is not fully overcomed after Edafence pre-treatment.

**Figure 4:**
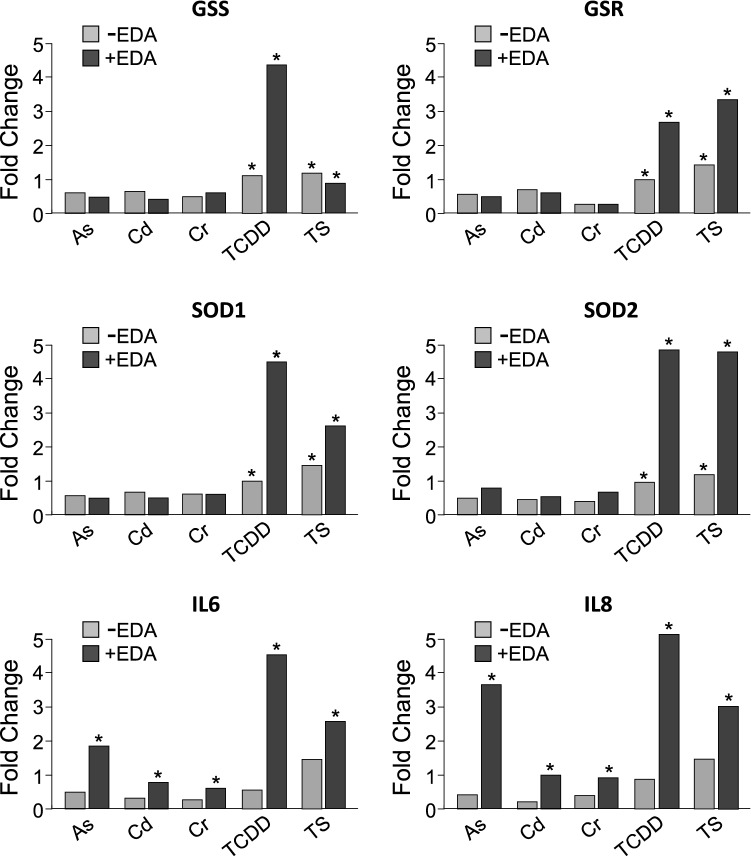
*Deschampia antarctica* aqueous extract (Edafence) promote homeostatic changes in the expression of key genes of the redox balancing and IL pro-immflamatory systems after acute exposure of Human Skin Organ Cultures to common ambient pollutants. Quantitative analysis of GSS, GSR, SOD1, SOD2, IL-6 and IL-8 gene expression by qRT-PCR in HSOC samples exposed to As(III), Cd(II), Cr(VI), 2,3,7,8-tetrachlorodibenzo-p-dioxin (TCDD) or Tobaco Smoke culture medium infusions (TS) pre-treated or not with *Deschampia antarctica* aqueous extracts (EDA). qRT-PCR data were analysed by a comparative C_T_ method, using 18S rRNA expression as internal control. Gene expression fold changes were represented as the ratio between means of 2^−ΔCt^ values in ambient pollutant exposed samples with respect to control samples in triplicates from two independent experiments (*, significant *p* ≤ 0.05).

As a rule, a major consequence of toxic xenobiotic compound exposure/accumulation in mammalian tissues is the induction of a sustained, detrimental oxidative stress induced by an abnormal generation of ROS which is closely associated with changes in the transcriptional activity of redox balancing system genes^8,17,24,31,54^. In this regard, here we have also quantified the expression level of key redox genes, including GSS, GSR, SOD1 and SOD2, in response to the acute exposure of HSOC samples to ambient polluntants. The transcriptional actiivity of these genes is extremely sensitive to changes in intracellular ROS levels^55^. We have found that the expression of all these genes is significantly increased after exposure to TCDD and TS, but not after exposure to toxic metals (Fig. 4). Notably, HSOC pre-treatment with Edafence strongly potentiates the transcriptional activation of these target genes (except for GSS in TS samples) after acute HSOC exposure to TCDD and TS, showing no effects after exposure to toxic metals (Fig. 4). These results suggest that the redox balance is impaired in the HSOC samples after acute exposure to TCDD and TS, requiring a homeostatic adjustment in the transcriptional network that regulates the intracellular redox balance. This redox response is further fostered by aqueous leaf extracts. Finally, we have also quantified the gene expression level of two pro-inflammatory cytokines, IL-6 and IL-8, directly involved in the response of human skin to severe external damage and subsequent wound healing^56–58^. Notably, we have found that pre-treatment of HSOC samples with *Deschampia antarctica* aqueous leaf extracts before acute exposure to ambient pollutants promotes the transcriptional activation of these genes in response to the severe injury induced by these toxic compounds (Fig. 4).

Overall, the results reported here indicate that a very short exposure (24 h) of HSOC samples to moderately high concentrations of common ambient pollutants, including As, Cd, Cr, TCDD and TS, is sufficient to induce a noticeable and severe tissue damage. In the case of toxic metals, the major morphological alteration observed was an extensive epidermal-dermal detachment particularly between the rete ridges and subjacent papillary dermis (Fig. 1A). This kind of lesion may be considered as an early indicator for the subsequent development of ulcerative/inflammatory processes in the tissue, in agreement with the previously reported effects of an acute/chronic exposure of human skin to As, Cd or Cr^6,10,18,22,24^. On the other side, desquamation of the stratum corneum was recurrently observed in HSOC samples after an acute exposure to TCDD (Fig. 1A), suggesting an impairment of the differentiation process of epidermal keratinocytes in the transit from basal to upper layers, in agreement with previous reports^34,35^. Notably, acute exposure of HSOC samples to the TS infusions used in this work resulted in a complete detachment of epidermal and dermal skin layers with and inflammatory infiltrate and slight edema in the epidermis (Fig. 1A). To our knowledge, this devastating lesion has not been previously described after acute or chronic exposures of human skin to tobacco smoke in different experimental models and clinical cases^37,39,41,42^. Probably, this effect results from the fact that concentrations of multiple toxic compounds in our TS infusions exceed a critical threshold for tissue integrity, and reflects the extremely poisonous nature of tobacco smoke for human skin.

Significantly, the effects on tissue morphology and on key tissue functional and structural parameters, including cell death (TUNEL) and proliferation (KI67) rates, and expression of the E-cadherin epidermal cell–cell adhesion protein, are different for each ambient pollutant (Figs. 1B and 2), suggesting that the molecular mechanisms initially affected by the acute pollutant exposure are diverse and probably implicating different networks of biochemical processes. In any case, it is to note that, except from TS infusions, acute exposure of HSOC samples to toxic pollutants promoted the emergence of early signatures of tumour progression and metastatic processes in epithelial tissues, e. g., excessive/abnormal cell proliferation rates and/or the loss of E-cadherin (Figs. 1B and 2A)^59,60^. These observations suggest that very short exposures of HSOC samples to ambient pollutants may be sufficient to promote sustained premalignant alterations in the skin, and are in line with previous studies reporting the carcinogenic potential of acute and chronic exposures of different human tissues to these toxic compounds^8,10,14,17,24^. Interestingly, no significant increases in the number of dead cells were observed after acute exposure of HSOC samples to toxic pollutants, again with the exception of TS infusions (Fig. 2B). As apoptosis is in many cases and anticancer mechanism and the deregulation/inhibition of apoptotic machinery is a hallmark of tumour progression^61^, this observation further supports the notion that acute exposures of skin to toxic pollutants may rapidly promote premalignant lesions. The marked increase in cell death rates concomitant to the decrease in cell proliferation rates in HSOC samples exposed to TS infusions strongly indicates, as mentioned above, the extreme harmful effect in the tissue of this toxic mixture. Besides these observations, it is evident that extensive additional work, out of the scope of this report, is required for an in deep dissection of the molecular mechanism involved in the rapid detrimental action of each ambient pollutant in the skin.

It is noteworthy that the acute exposure of HSOCs to all toxic compounds tested here strongly induced the cytoplasmic accumulation and nuclear translocation of AhR (Fig. 3), suggesting the systemic activation of the transcriptional network involved in the response against the exposure to xenotoxic compounds^29,32^. These results are in close agreement with previous reports in different experimental systems showing that exposure of mammalian cells to As, Cd and Cr^62,63^, dioxins^31,64^ and tobacco smoke^41,65^ directly modulate the expression and activity of AhR-regulated genes and AhR-dependent drug metabolizing enzymes. The AhR network is particularly sensitive to changes in the redox state of the mammalian cell and is associated with a rapid response to the oxidative stress induced by xenobiotics/ambient pollutants^31,66^, considered as a critical factor in environmental-induced carcinogenesis in human ecosystems^67^. In this sense, it has been widely reported that a recurrent effect of the exposure to As, Cd, Cr, TCDD or TS is the abnormal generation of ROS and subsequent increase of cellular oxidative stress^17,67–72^. Here we have also analyse the expression of four central genes of the redox system, GSS, GSR, SOD1 and SOD2, whose expression is extremely sensitive to changes in intracellular ROS levels^55^, in response to the acute exposure of HSOC samples to ambient polluntants. Intriguingly, a significant activation of these genes is only observed in HSOC samples exposed to TCDD or TS infusion, but not to toxic metals (Fig. 4). As previously mentioned, it is widely accepted that oxidative stress and abnormal ROS generation play critical roles in the toxic effects of acute and chronic exposures of mammalian cells to As, Cd or Cr. However, it has been also reported that the dynamics of abnormal ROS production and subsequent induction of oxidative stress may be an indirect and delayed process, requiring the inhibition of different targets in the redox system^17,70,71^. This fact may explain the lack of significant changes in the expression of GSS, GSR, SOD1 and SOD2 in HSOC samples after very short exposures to toxic metals.

The results reported here also suggest that the aqueous leaf extracts of *Deschampsia antarctica* (Edafence) may have a significant protective effect against the exposure of human skin to the ambient pollutants tested here. Thus, we have shown that Edafence pre-treatments efficiently prevent the morpho/structural alterations induced by the acute exposure of HSOC samples to these toxic compounds, restoring the expression and localization of E-cadherin in the epidermis and counterbalancing the alterations in cell proliferation and cell death ratios (Figs. 1 and 2). The biochemical mechanisms implicated in this prominent protective effect deserves further investigations. However, it is tempting to speculate that an effective defusing of the oxidative stress induced by the exposure of HSOC samples to these toxic compounds plays a critical role in the process. For one side, *Deschampsia antarctica* aqueous extracts present high amounts of antioxidant compounds, mainly phenolic acids and flavonoids^50–53^. For the other side, Edafence, may directly or indirectly modulate the expression of AhR and key genes of the redox system in response to the exposure of HSOC samples to toxic ambient pollutants (Figs. 3 and 4). These observations suggest that the potential ROS scavenging activity of Edafence is the key factor preventing skin tissue damage. In addition, Edafence pre-treatment of HSOC samples triggers the expression of the pro-inflammatory cytokines IL-6 and IL-8 (Fig. 4), involved in the activation of wound healing and the first steps of skin regeneration, a physiological process that depends on a controlled ROS production^73^. As a whole, these results suggest that the natural aqueous extract of Deschampsia antarctica (Edafence) might be effectively used in vivo to protect human skin routinely against the daily exposure to ambient pollution.

## Methods

### Establishment of human skin organ cultures (HSOC)

The adult human skin Organ Culture (HSOC) provides a useful model to study the molecular mechanisms on skin repair and allows the evaluation of new therapies and products, avoiding the use of in vivo animal models. Human skin samples of about 1 cm^2^ area were taken from volunteer donors, typically 40–60 years old women exhibiting normal skin of phototype II–III in Fitzpatrick classification, as plastic surgical skin remnants. Eligible patients provided written informed consent, and the Ethical committee of the Ramon y Cajal University Hospital approved this procedure. All methods were performed in accordance with the Spanish Government and UE relevant guidelines and regulations regarding experimentation with human samples.

To perform tissue cultures, skin samples were transferred to the laboratory, and the adipose tissue was removed using a scalpel. Then, skin explants were cut with full thickness, using a scalpel, in pieces of 50–70 mm^2^, and were washed 3 times in phosphate buffer solution (PBS). Then, skin pieces were soaked in DMEM medium supplemented with 10 × P/S and Amphotericin B, for 30 min. Subsequently, half of the skin dermal part was submerged in 2.5 mg/ml Type I Bovine Collagen gels. To that end, equal volumes of Type I Bovine Collagen Solution Pre-Neutralized in DMEM/F-12 Medium (PureCol Ez gel, 5 mg/ml, Advanced Biomatrix), and 2 × supplemented DMEM medium (2 × P/S and Amphotericin B, 20% FBS, 20 µg/ml Insulin, 20 ng/ml hydrocortisone and 4 mM glutamine) were mixed at 4 °C, using pre-cooled pipet tips and tubes on ice.

To perform the HSOCs, 300 or 500 µl of 2.5 mg/ml Type I Bovine Collagen DMEM/F-12 solution was added to each well (24 or 12 multiwell plate, respectively) and skin samples were placed in the collagen, facing epidermis upwards. The multiwell plate was transferred to a 37 °C incubator with 5% CO_2_ humidified atmosphere for 30 min to 2 h, to allow the polymerization of the gels with the skin pieces and 100–200 µl of supplemented DMEM culture medium was added to each well, enough to cover the rest of the dermal part of skin pieces, and the epidermis was left exposed to air–liquid interface. DMEM medium was supplemented with 1 × P/S and Amphotericin B, 10% FBS, 10 µg/ml Insulin, 10 ng/ml hydrocortisone and 2 mM glutamine and changed every day.

### Preparation tobacco smoke extract infusions

Tobacco smoke extract infusions in culture medium were obtained as previously described with slight modifications^74^. Briefly, a filter cigarette (Ducados brand; Imperial Tobacco) was attached to the lateral connection of a vacuum flask, and a negative pressure pump was applied to the upper opening of the flask. Then the cigarette was lit and the smoke drawn making bubbling in the vacuum flask containing DMEM medium without phenol red. The vacuum flask was further placed on a magnetic stirrer in order to dissolve the maximum amount of tobacco smoke in the DMEM medium. A stock 100% TS infusion was performed lighting 20 cigarettes in 20 ml. The average duration of the combustion of each cigarette was 7 min. A working TS infusion was pepared by diluting to a 20% in DMEM medium. The detailed composition of the Ducados tobacco smoke has been previously reported^75^.

### Acute expossure of HSOC to toxic ambient pollutants and pre-treatments with *Deschampsia antarctica* aqueous extracts

*Deschampsia antarctica* leaf samples were harvested from plants identified, cultivated and supervised in a controlled envinronment by Cantabria Labs Experimental Department. Dry green leaves were milled and extracted by percolation with water at 40–60 °C, during 4–6 h. The extract was filtered through a 1 μm filter and lyophilized. Working samples were provided as powder of desiccated aqueous leaf extracts (20–30% w/w) adsorbed in corn (*Zea mays*) starch (70–85% w/w). A comprehensive analysis of the bioactive compounds contained in *Deschampsia antarctica* aqueous extracts has been recently reported^53^. It is to note the high content of flavonoids and derivatives, mainly Orientin, Isoweriajaponin 2″-O-beta-arabinopyranoside, Coumesterol, Tricin as well as hydroxycinnamic acids, including p-Coumaroyl glycolic and tartaric acids, and Caffeic acid glucoside. For reasons of intellectual property protection, a voucher specimen of this material has not been yet deposited elsewhere in a publicly available herbarium, but is available upon request to Cantabria Labs.

Working concentrations for toxic compounds were previously established in human skin primary cells, fibroblasts and keratinocytes as described^76,77^. For the preparation of working solutions of the different toxic compunds, 9 mM of NaAsO_2_ (As(III), arsenic), 0,3 mM of CdCl_2_ (Cd(II), Cadmium), 0.5 mM of CrO_3_ and CrCl_3_, at equal concentrations, (Cr(III/VI), Chromium), 10 nM of 2,3,7,8-Tetrachlorodibenzo-p-dioxin (TCDD) or dilutions of Tobacco Smoke (TS) extract infusions, were dissolved in supplemented DMEM culture medium and filtered through a 0.2 µm pore membranes. For acute exposure to toxic working solutions, HSOC samples were incubated in each particular DMEM solution, for 24 h in the case of As(III), Cd(II), and Cr(VI) & Cr(III) and TSE or for 3 h in the case of TCDD. For pre-treatments with *Deschampsia antarctica* aqueous extracts, HSOC samples were incubated in Edafence working solutions of for 24 h before to the exposure to toxic compunds. For Edafence working solution preparation, 10 mg/ml of Deschampsia antarctica extract absorbed on starch, was resuspended in agitation for 30 min in DMEM supplemented media at a final concentration of 2.5 mg/ml, and then filtered through a 0.2 µm pore membranes. After toxic exposure, with or without Edafence pre-treatments, HSOC samples were washed in fresh DMEM medium, further growth to reach a total of 48 h of culture from the start of the toxic exposure and harvested for for histological, immunohistochemical and gene expression analysis.

### Histological and Immunohistochemical analysis

For histological and immunohistochemical analysis, selected parts of HSOC samples were fixed with 3.7% aqueous formaldehyde solution and embedded in paraffin following standard procedures. Histological sections of 8–10 µm thickness were stained with H&E for morphological analysis or permeabilized in 0.1% Triton X-100 for 30 min at RT and subsequently incubated o/n at 37 °C for 2 h with the corresponding primary antibodies for protein immunolocalization in the tissue as previously described in detail^78,79^. Primary antibodies used include mouse monoclonal anti human E-cadherin (Santa Cruz), rabbit monoclonal anti human KI-67 (Abcam) and rabbit monoclonal anti human AHR (Merck). For apoptotic cell death in skin sections, the TUNEL detection kit (Roche) was used according to the instructions of the manufacturer. Image acquisition was performed using a Nikon Eclipse Ci coupled to a Jenoptik PROGRES GRYPHAX® SUBRA Super HD camera and suited Version 1.1.8.153 image software pack.

For histological analysis of H&E-stained sections, different standard morphological parameters were evaluated in treated as compared to control HSOC samples, including overall tissue integrity, epidermal thickness, loss of epidermal papillae, inflammation, fibroplasia and hyperpigmentation. For quantifications of positive cell ratios for the indicated proteins, the number of positive cells in 8–10 µm histological sections was determined using HD FiJI software processed images. For each particular sample, positive cells were counted in three different 300 × 300 µm square areas, encompassing half/half epidermal and dermal regions, and the corresponding positive cell average (%) of the sample was calculated. The mean + SD of the positive cell average of at least three different samples for each experimental condition was further determined. Comparisons between groups were performed by Student’s t test using the SPSS 15.0 software.

### Quantitative gene expression analysis

For RNA extraction, RNeasy mini kit and RNase-Free DNase Set (both from Qiagen) were used. Selected parts of HSOC samples were homogenized using TriPure™ isolation Reagent (Roche), disaggregated and processed using scissors and a Polytron® homogenizer (PT 1200 E, Kinematica). For reverse transcription, MLV enzyme (Promega) was used, loading the same amount of RNA. qRT-PCR assays were performed for gene expression analysis, using Power SYBR Green (Applied Biosystems), following manufacturer’s instructions. Specific primers for quantitative transcript detection are annotated below and were designed among different exons thereby avoiding residual genomic DNA amplification:

GSS: Fwd 5′-CCAAGACCGAAGGCTGTTTGTG-3′; Rev 5′- TGTGACCTCTCCAGCAGTAGAC-3′.

GSR: Fwd 5′-TATGTGAGCCGCCTGAATGCCA-3′; Rev 5′- CACTGACCTCTATTGTGGGCTTG-3′.

SOD1: Fwd 5′-CTCACTCTCAGGAGACCATTGC-3′; Rev 5′- CCACAAGCCAAACGACTTCCAG-3′.

SOD2: Fwd 5′-CTGGACAAACCTCAGCCCTAAC-3′; Rev 5′-AACCTGAGCCTTGGACACCAAC-3′.

IL-6: Fwd 5′-CCGGGAACGAAAGAGAAGCT-3′; Rev 5′-GCGCTTGTGGAGAAGGAGTT-3′.

IL-8: Fwd 5′-CTTTCCACCCCAAATTTATCAAAG; Rev 5′-CAGACAGAGCTCTCTTCCATCAGA-3′.

For statistical analyses of gene expression data, an unpaired t-test was applied, setting p ≤ 0.05 and fold change ≥ 2.0 as limits for significance. qRT-PCR data were analysed by a comparative C_T_ method, using 18S rRNA expression as internal control. qRT-PCR data were analysed by a comparative C_T_ method, using 18S rRNA expression as internal control. Gene expression fold changes were represented as the ratio between means of 2^-ΔCt^ values in ambient pollutant exposed SOC samples with respect to control samples.
